# Just Breathe

**Published:** 1906-07

**Authors:** H. H. Way

**Affiliations:** St. Thomas, Ontario.


### "JUST BREATHE."

H. H. WAY, D. D. S.; L. D. S. ST. THOMAS, ONTARIO.

I am beginning to more fully realize that our indoor professional people are too reckless of our own health. Our very environments are conducive to it. We forget our own, in the daily routine of serving others.

A valued English authority, Dr. A. L. Schofield has just placed "Good Air" in his five laws of health, and I thank him for it though I may possibly flavor of the crank in doings so.

I believe myself but one of a rapidly increasing number of living proofs of the several benefits of "deep abdominal breathing," as a habit.

The seeker after better air, and more of it, does not necessarily leave his own home and occupation to find it. It is the most accessible blessing in all this lower world of ours: nor is it likely to be "cornered" for some time yet.

I need not go into this idea of a perfect acration, only strive to centralize your attention sufficiently long enough to form the easy breathing habit.

"Breathe the best air you can get, and Plenty of it." In due time you will experience some wholesome revelations. Its about the only effective remedy for the white plague of civilization. You will feel the renewed life all the way from "that little fillip to the heart's action;" the glow of an increased circulation; the excreting organs working freely with a future freedom from those dreaded troubles of indigestion and constipation.

"When you walk the streets, contract and extend the muscles in one part of the body after another, — not conspicuously, but with force."

Be always sure that you sleep in a room with some out door communication and you will seldom have "a cold."

Some one has already said that "it is a sin to be sick;" so, if you are still not feeling right look still deeper into your daily habits, and you will find the lurking enemy.

